# The Effect of a Mobile and Wearable Device Intervention on Increased Physical Activity to Prevent Metabolic Syndrome: Observational Study

**DOI:** 10.2196/34059

**Published:** 2022-02-24

**Authors:** Hee Jin Kim, Kang Hyun Lee, Jung Hun Lee, Hyun Youk, Hee Young Lee

**Affiliations:** 1 Department of Emergency Medicine Yonsei University Wonju College of Medicine Wonju Republic of Korea

**Keywords:** mHealth, physical activity, wearable device, metabolic syndrome, health care, exercise, intervention, Asia, Korea, rural

## Abstract

**Background:**

Research on whether wearable devices and app-based interventions can effectively prevent metabolic syndrome (MetS) by increasing physical activity (PA) among middle-aged people living in the rural areas of South Korea remains insufficient.

**Objective:**

The aim of this study was to determine whether mobile and wearable device interventions can improve health indicators, including PA, in MetS risk groups in rural South Korea.

**Methods:**

In this clinical trial, performed from December 2019 to June 2020, participants were asked to use a wearable device (GalaxyWatch Active1) alone (standard intervention) or the wearable device and mobile app (Yonsei Health Korea) (enhanced intervention). Clinical measures and International Physical Activity Questionnaire (IPAQ) scores were evaluated initially and after 6 months. The number of steps was monitored through the website. The primary outcome was the difference in PA and clinical measures between the enhanced intervention and standard intervention groups. The secondary outcome was the decrease in MetS factors related to the change in PA.

**Results:**

A total of 267 participants were randomly selected, 221 of whom completed the 6-month study. Among the 221 participants, 113 were allocated to the enhanced intervention group and 108 were allocated to the standard intervention group. After 6 months, the body weight and BMI for the enhanced intervention group decreased by 0.6 (SD 1.87) and 0.21 (SD 0.76), respectively (*P*<.001). In both groups, systolic blood pressure, diastolic blood pressure, waist circumference, and glycated hemoglobin A_1c_ (HbA_1c_) decreased (*P*<.001). The total PA was approximately 2.8 times lower in the standard intervention group (mean 44.47, SD 224.85) than in the enhanced intervention group (mean 124.36, SD 570.0). Moreover, the enhanced intervention group achieved the recommended level of moderate to vigorous physical activity (MVPA), whereas the standard intervention group did not (188 minutes/week vs 118 minutes/week). Additionally, the number of participants in the enhanced intervention group (n=113) that reached 10,000 daily steps or more after the intervention increased from 9 (8.0%) to 26 (23.1%) (*P*=.002), whereas this number did not increase significantly in the standard intervention group (n=108), from 8 (7.4%) to 16 (14.8%) (*P*=.72). The number of participants without any MetS factors increased by 12 (11%) and 8 (7%) in the enhanced and standard intervention group, respectively.

**Conclusions:**

PA monitoring and an intervention using wearable devices were effective in preventing MetS in a rural population in Korea. Blood pressure, waist circumference, and HbA_1c_ were improved in both intervention groups, which were effective in reducing MetS factors. However, only the participants in the enhanced intervention group continuously increased their MVPA and step counts above the recommended level to prevent MetS. Body weight and BMI were further improved, and a higher number of participants with zero MetS factors was attained from the enhanced intervention.

**Trial Registration:**

Clinical Research Information Service KCT0005783; https://cris.nih.go.kr/cris/search/detailSearch.do/16123

## Introduction

### Background

Metabolic syndrome (MetS) is a disease characterized by three or more of the following five factors: abdominal obesity, high blood pressure, high blood sugar, high triglycerides, and high high-density lipoprotein cholesterol [1]. Approximately one-quarter of the world’s adult population has MetS [2], which is a major cause of disability as well as a leading cause of death in 60% of the global population [3]. MetS is also a serious risk factor for heart disease, stroke, and type 2 diabetes [4]. Research shows that rural residents are less accessible and active than urban residents, who reported exercising in constructed environments such as neighborhood streets, parks, and shopping malls. There is also a difference in income level between urban and rural populations [5]. Moreover, studies have shown that rural residents lack education regarding proper eating habits compared to urban residents and are more likely to be obese; in addition, their socioeconomic status and access to medical services are lower than those of urban residents [6,7]. Rural residents appear to have a higher risk for MetS and a higher disease burden than urban residents due to these differences in infrastructure [8].

Global smartwatch sales continue to increase, which are expected to reach 109.2 million units in 2023 [9], and the wearable device market is expected to continue to expand [10]. Mobile health (mHealth) technologies using apps and wearable devices are becoming increasingly popular, as they allow patients to monitor their own health conditions [11,12]. Moreover, mobile apps suggest a variety of methods to prevent disease and maintain and improve patient health [13]. Wearable devices can improve the lifestyle of patients with chronic diseases [14]. Previous reviews on promoting physical activity (PA) in adults have shown evidence for the effectiveness of mHealth on increasing PA [15,16]. Most health-related behaviors such as eating well and exercising regularly can lead to significant improvements if sustained through motivation [4,17]. However, it is difficult to promote the self-management of chronic diseases among the elderly, which is also a valid concern among the elderly population in Korea [14]. According to a study by the Korea Institute for Health and Social Affairs, which measured the PA of Korean adults (N=697), the proportion of men aged 65 years or above who were engaged in vigorous PA decreased by approximately half from 9.3% in 2010 to 4.9% in 2018, and the proportion of those engaged in moderate PA decreased from 14.6% to 10%. During the same period, the proportion of women of the same age engaging in vigorous PA decreased from 3.3% to 2% and the proportion engaging in moderate PA decreased from 6.6% to 4% [18]. These results suggest that lack of PA might be a health risk factor for adults in Korea [19].

Previous studies have shown that PA offers a variety of health benefits, including reducing anxiety and depression; improving sleep and quality of life; and lowering the risk of developing diabetes, heart disease, and many cancers [20]. It has been reported that lack of PA increases health risks, including coronary heart disease, type 2 diabetes, and cancer, and shortens life expectancy from major noncommunicable diseases [19,20]. Regular PA can help to prevent aging-related declines in physical function, and reduces morbidity and mortality [21]. Since self-management of chronic diseases requires treatment or behavioral modification, support tools are needed to maintain practice in daily life [22]. Interventions through wearables and/or smartphone apps are effective in promoting PA in the adult population [23].

### Objectives

We hypothesized that intervention components, especially wearable devices and mobile apps, for the prevention of MetS will have a positive effect on PA in the middle-aged population in Korea. The purpose of the study was two-fold: (1) to compare the changes in clinical values and PA between an enhanced intervention group and standard intervention group that had not received the intervention for 6 months, and (2) to objectively reduce risk factors of MetS. We further explored whether the change in MetS risk factors is related to the measured PA change.

## Methods

### Ethical Considerations

The study was performed at Yonsei University Wonju Severance Christian Hospital (Wonju, Korea) between December 2019 and June 2020, and was approved after deliberation by the Research Ethics Review Committee (approval number CR319089; trial registration number KCT0005783). Participants of the clinical trial were informed of the purpose and procedure of the study through the consent form for participation in the study and were asked to fill out the consent form in writing. Instructions were provided in the questionnaire, including statements that no personal information will be exposed for purposes other than research, and that participation is voluntary and can be withdrawn at any time.

### Inclusion and Exclusion Criteria

**Figure 1:**
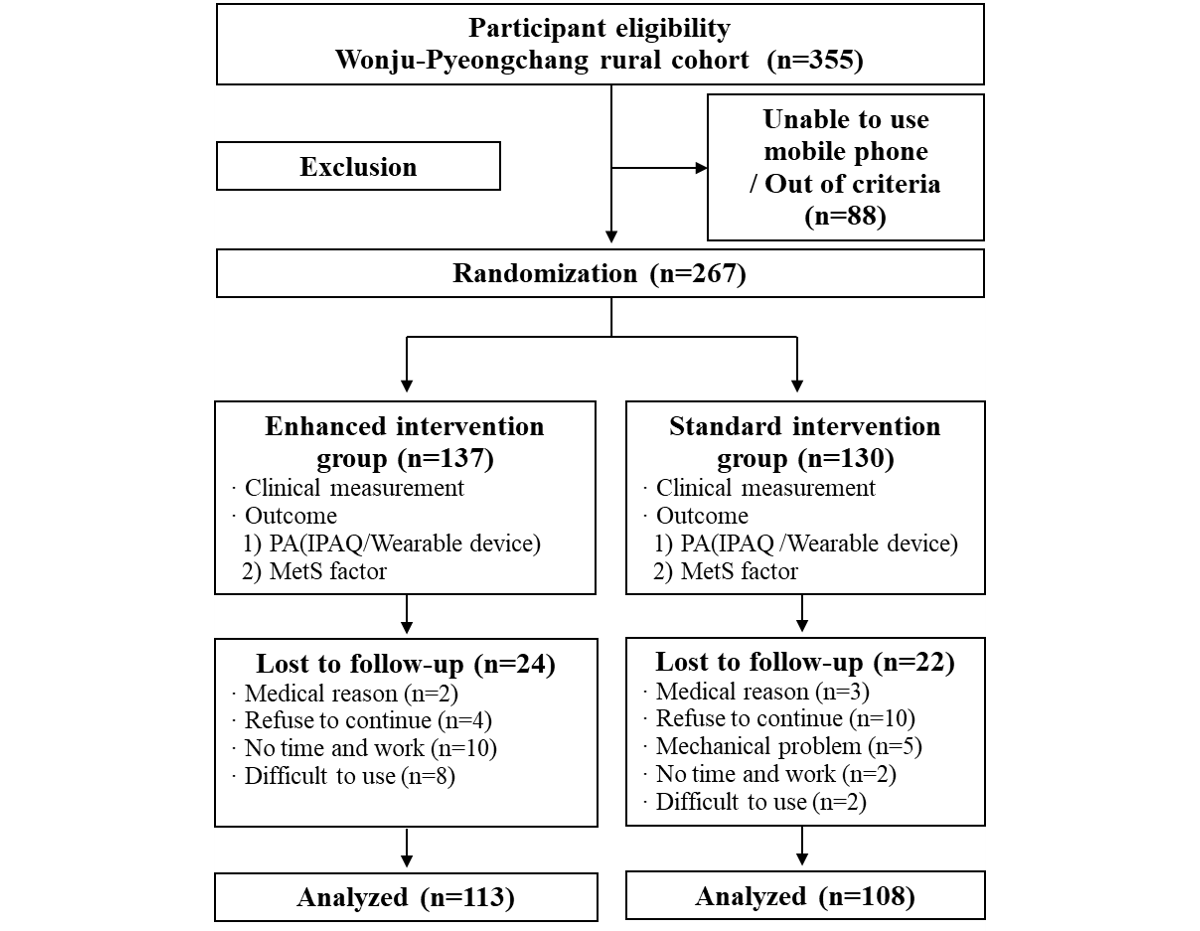
Flowchart of study participants. PA: physical activity; IPAQ: International Physical Activity Questionnaire; MetS: metabolic syndrome.

The inclusion criteria were as follows: (1) adults aged 40 to 80 years, (2) people with one or more MetS factors (Textbox 1), (3) participating in the Wonju-Pyeongchang cohort study via the Korea Centers for Disease Control and Prevention, (4) agree to participating in the clinical trial, and (5) able to participate after understanding the training and instructions. A diagnosis of MetS was based on the diagnostic criteria for MetS in Korea, modified from the National Cholesterol Education Program Adult Treatment Panel-III criteria. A total of 221 people (113 in the enhanced intervention group and 108 in the standard intervention group) were enrolled according to the standards for randomized controlled trials (Figure 1).

Besides having a MetS diagnosis or risk factor, participants also had to have a smartphone using the Android operating system. Participants had to be able to receive and read text messages, without have any difficulty in using wearable devices and mobile apps that will send alerts. Some patients were excluded 1 month before participation due to the use of warfarin (eg, Coumadin); additionally, those with physical disabilities who could not use a wearable device, those with skin diseases and dysfunction, and those who had an aversion to the wearable device were excluded.

**Metabolic syndrome factors considered for study inclusion.**

- Waist circumference ≥90 cm in men and ≥80 cm in women
- Systolic blood pressure ≥130 mmHg or diastolic blood pressure ≥85 mmHg
- Triglyceride level ≥150 mg/dL
- High-density lipoprotein cholesterol level <40 mg/dL in men and <50 mg/dL in women
- Fasting plasma glucose level ≥100 mg/dL

### Measures

Participants were selected among the existing Wonju-Pyeongchang rural cohort list, and only those who were connected and willing to participate in the clinical study were recommended to visit the Smart Healthcare Support Center of Wonju Severance Christian Hospital. On the first visit, an approximate 1-hour session was performed including clinical measurements, MetS education, and device education. Clinical measurements comprised an 8-hour fasting blood test. Weight and height were measured using an automated digital scale (Tanita T6360) with shoes and jackets removed. Participants were measured in a fasting state without outerwear. BMI was calculated as the ratio of weight to height squared. Waist circumference was measured using a digital tape measure (PIE, Bagle Labs Co, Korea). An automated blood pressure meter (HEM-9000T, Omron Co, Japan) was used to measure diastolic/systolic blood pressure. All participants underwent clinical examination before and after the start of the study.

### Intervention

**Figure 2:**
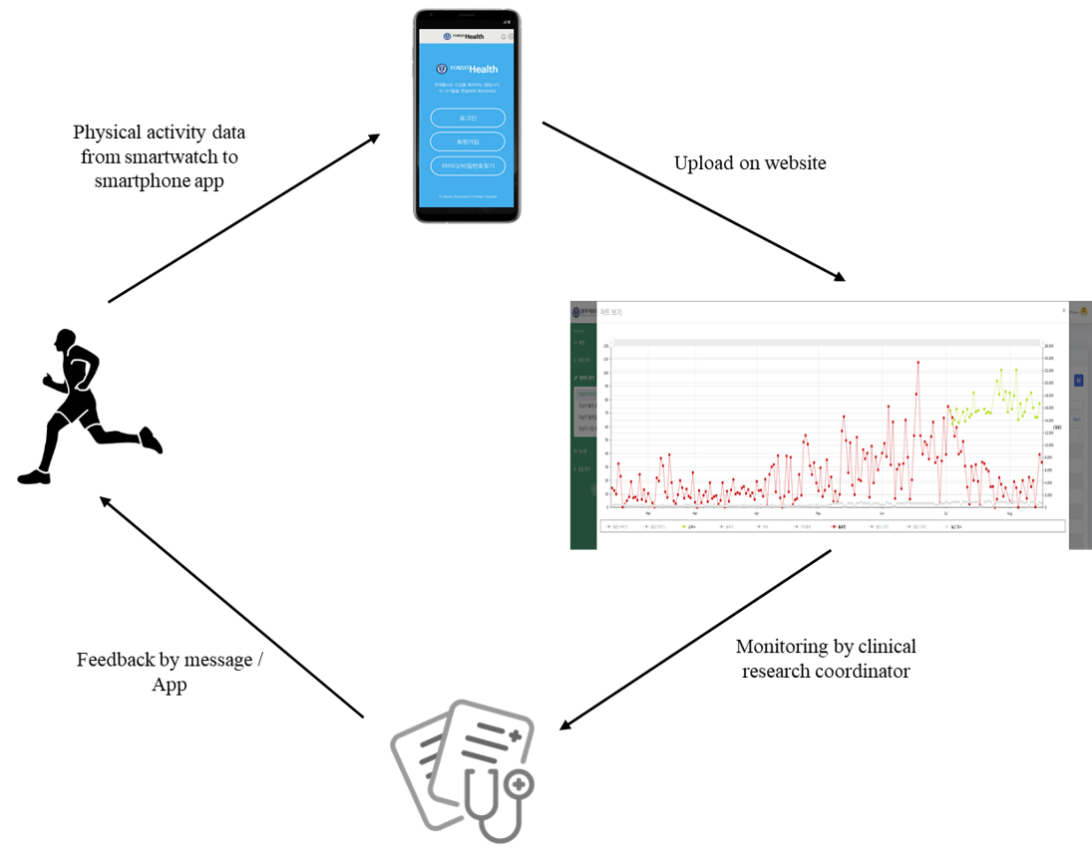
Flow of intervention.

The structure and contents of the mobile app and text messages were developed by the researchers and clinical research coordinators. The National Health Insurance Service, the guidelines for endocrinology, educational materials, and literature for existing MetS programs were referenced. The overall flow chart of the study is shown in Figure 2. A wearable device (Galaxy Watch Active1) was provided to both the enhanced and standard intervention groups. The participants in the enhanced intervention group were further provided with a mobile app (Yonsei Health) that allows users to check text messages, phone contacts, videos, and reference educational materials customized for the prevention and treatment of MetS. Participants were recommended to use the provided mobile app and wearable device at least 3 times a week (more than 8 hours/day). The data from the wearable device were transmitted to the web server through the website where the measurements were checked by a research nurse. On the website, changes in PA could be observed as the accumulated number of patients’ steps, and an SMS text message could be sent to the patient directly. The standard intervention group received no intervention other than self-monitoring, whereas the enhanced intervention group was provided with feedback on their PA. The feedback comprised reward messages for increased activity or sufficient PA, and encouraging messages were provided every other week if the level of activity decreased or remained unchanged compared with that of the previous week. The feedback was centered on encouraging and maintaining PA. Health information provided through the mobile app contained guidance on PA and lifestyle to prevent or reduce risk factors for MetS, mainly comprising medical, lifestyle, nutrition, exercise, and cognitive categories.

### Evaluation

#### Data Collection

The evaluation was performed at baseline and 6 months later. Demographic data such as age, gender, education level, and average monthly income were collected face to face using questionnaires. Clinical measures were collected using the same parameters to ensure comparability before and after the study.

#### PA Assessment

The short format of the International Physical Activity Questionnaire (IPAQ) is a form for population surveillance and the long format is designed to be used in research that requires information on various PA areas [24]. The long-form IPAQ includes a total of 31 items, providing a more detailed assessment of the level of PA than the short format [25]. The questions include home and garden work activities, occupational activities, transportation use, and leisure time in the last 7 days, which are designed to record the time of walking, vigorous and moderate PA, and time spent sitting or lying down. The questionnaire was prepared by interviewing the research coordinator and constructing a self-written form. Metabolic equivalents of task (MET) was used to calculate official scores, which is an intensity unit of PA determined as the ratio between the metabolic rate during a given activity over the resting metabolic rate [26]. PA was represented using the number of days and duration (time) of activity, and was calculated based on the following formulas [27]:

Walking (MET hours/week) = 3.3 × walking hours × days **(1)**

Moderate PA (MET hours/week) = 4.0 × moderate PA hours × days **(2)**

Vigorous PA (MET hours/week) = 8.0 × vigorous PA hours×days **(3)**

Total MET minutes/week = sum of Walking + Moderate + Vigorous MET minutes/week scores **(4)**

#### Step Counts

The number of steps was measured using a wearable device. After each measurement, data were automatically uploaded to the website through a Bluetooth-connected smartphone app. The wearable device used in this study was a smartwatch that enabled Bluetooth connection with the Android operating system. This device was adopted because the user interface and method of use were similar to those of smartphones; therefore, there was no difficulty for participants to use it.

### Statistical Analysis

The general characteristics of the subjects are summarized using descriptive statistics. A paired *t* test was used to compare clinical measures before and after the intervention, and a *t* test and a *χ^2^* test were performed to compare differences between groups. SPSS 26.0 was used for statistical analysis; *P*<.05 was considered significant.

## Results

### Participant Characteristics

Participants were recruited after obtaining Institutional Review Board approval in December 2019, and registration began thereafter. Data collection lasted for up to 6 months from the date of registration. Finally, of the 221 subjects included in the study, 113 were allocated to the enhanced intervention group and 108 were allocated to the standard intervention group. In both groups, more than half of the participants had secondary or higher education (80.9% and 81.4%, respectively), and the mean age was 64.8 (SD 6.3) and 66.3 (SD 6.2) years in the enhanced and standard intervention group, respectively (Table 1). Women accounted for more than 50% of the cohort. The two groups did not differ with respect to educational attainment, marital status, and monthly income. Clinical measures such as BMI, glycated hemoglobin (HbA_1c_), triglycerides, and blood pressure were not different between groups; however, high-density lipoprotein cholesterol levels differed between the two groups (*P*=.03). Additionally, the proportion of patients with MetS at baseline was higher in the standard intervention group (*P*=.03).

**Table 1:** Characteristics of the study participants at baseline.

Variables			Enhanced intervention group (n=113), n (%)		Standard intervention group (n=108), n (%)		*P* value	
**Age group (years)**								.07
	35-50	0 (0)		1 (0.9)				
	51-70	86 (76.1)		78 (72.9)				
	>70	27 (23.9)		29 (26.2)				
**Gender**								.21
	Male	45 (39.8)		52 (48.1)				
	Female	68 (60.2)		56 (51.9)				
**Level of education**								.34
	No response	0 (0)		1 (0.9)				
	No formal schooling	2 (1.8)		1 (0.9)				
	Elementary school	19 (16.8)		18 (16.7)				
	Middle school	23 (20.4)		20 (18.5)				
	High school	44 (38.9)		36 (33.3)				
	University	20 (17.7)		24 (22.2)				
	Postgraduate	5 (4.4)		8 (7.4)				
**Marital status**								.59
	Never married	1 (0.9)		0 (0)				
	Married	106 (93.8)		102 (94.4)				
	Divorced	1 (0.9)		0 (0)				
	Widowed	5 (4.4)		6 (5.6)				
**Average monthly income (Won^a^)**								.19
	<1 million	15 (13.3)		12 (11.1)				
	1-2.99 million	54 (47.8)		55 (50.9)				
	3-4.99 million	34 (30.1)		23 (21.3)				
	≥5 million	10 (8.8)		18 (16.7)				
**BMI (kg/m^2^)**								.61
	<23	22 (19.5)		16 (14.8)				
	23-24.9	37 (32.7)		34 (31.5)				
	≥25	54 (47.8)		58 (53.7)				
**HbA_1c_^b^ **(%)****								.11
	≤5.6	50 (44.3)		34 (31.5)				
	5.7-6.4	53 (46.9)		67 (62.0)				
	≥6.5	10 (8.8)		7 (6.5)				
**TG^c^ (mg/dL)**								.15
	<200	94 (83.2)		81 (75.0)				
	200-239	8 (7.1)		14 (13.0)				
	≥240	11 (9.7)		13 (12.0)				
**HDL-C^d^ (mg/dL)**								.03
	<60	90 (79.6)		94 (87.0)				
	≥60	23 (20.4)		14 (13.0)				
**SBP^e^ (mmHg)**								.32
	<120	9 (8.0)		6 (5.6)				
	120-139	51 (45.1)		38 (35.2)				
	140-159	39 (34.5)		50 (46.3)				
	≥160	14 (12.4)		14 (13.0)				
**DBP^f^ (mmHg)**								.60
	<80	12 (10.6)		13 (12.0)				
	80-89	44 (38.9)		41 (38.0)				
	90-99	45 (39.8)		40 (37.0)				
	≥100	12 (10.6)		14 (13.0)				
**MetS^g^ factors**							.03	
	1	16 (14.2)		10 (9.3)				
	2	35 (31.0)		25 (23.1)				
	3	32 (28.3)		33 (30.6)				
	4	26 (23.0)		33 (30.6)				
	5	4 (3.5)		7 (6.5)				

^a^10 Won=US $0.01.

^b^HbA_1c_: glycated hemoglobin.

^c^TG: triglyceride.

^d^HDL-C: high-density lipoprotein cholesterol.

^e^SBP: systolic blood pressure.

^f^DBP: diastolic blood pressure.

^g^MetS: metabolic syndrome.

### Change in Participant Measures From Baseline to Follow-Up

Table 2 shows the PA and clinical values measured before and after the intervention. In the enhanced intervention group, body weight, BMI, systolic and diastolic blood pressure, waist circumference, and HbA_1c_ decreased (*P*<.001), whereas in the standard intervention group, only systolic and diastolic blood pressure, waist circumference, and HbA_1c_ decreased (*P*<.001). In both groups, vigorous PA increased and sitting time decreased (*P*<.001), and total PA (*P*=.02 and *P*=.04 in the enhanced and standard intervention group, respectively) and time of walking (*P*<.001) also increased. However, in the enhanced intervention group, vigorous PA, total PA, and walking time increased more than those in the standard intervention group, and the sitting time decreased. The increase of moderate PA was also larger in the enhanced intervention group (Table 2).

**Table 2:** Change in clinical and physical activity outcomes in the study groups following the intervention.

Variables			Enhanced intervention group (n=113)									Standard intervention group (n=108)						
			Baseline		Follow-up		Change		*P* value		Baseline			Follow-up		Change		*P* value
Weight (kg), mean (SD)			65.5 (10.7)		64.9 (10.5)		–0.6 (1.8)		<.001		65.7 (9.0)			65.8 (9.0)		0.1 (3.09)		.55
BMI (kg/m^2^), mean (SD)			25.5 (3.0)		25.3 (2.9)		–0.2 (0.7)		<.001		25.7 (2.9)			25.7 (2.9)		0.0 (0.9)		.81
SBP^a^ (mm Hg), mean (SD)			139.5 (15.8)		126.5 (13.9)		–13.0 (14.6)		<.001		141.8 (18.1)			126.1 (21.4)		–15.6 (24.4)		<.001
DBP^b^ (mm Hg), mean (SD)			89.7 (8.6)		80.0 (9.0)		–9.7 (8.4)		<.001		89.1 (8.8)			79.8 (9.1)		–9.2 (8.1)		<.001
WC^c^ (cm), mean (SD)			91.2 (7.6)		87.0 (8.0)		–4.1 (4.4)		<.001		92.4 (6.7)			89.5 (7.7)		–2.9 (5.0)		<.001
HDL-C^d^ (mg/dL), mean (SD)			50.5 (10.1)		49.6 (10.6)		–0.8 (7.9)		.25		47.5 (10.7)			48.5 (10.4)		0.9 (7.0)		.17
TG^e^ (mg/dL), mean (SD)			146.6 (88.1)		155.2 (99.5)		8.5 (63.3)		.15		170.4 (150.8)			150.1 (87.0)		–20.3 (134.5)		0.11
HbA_1c_^f^ (%), mean (SD)			5.7 (0.4)		5.6 (0.4)		–0.1 (0.2)		<.001		5.8 (0.6)			5.7 (0.5)		–0.1 (0.2)		<.001
**PA^g^, mean (SD)**																		
	Vigorous PA (hours/week)	0.8 (2.3)		2.3 (4.8)		1.5 (5.3)		<.001		0.8 (1.6)			1.5 (2.2)		0.7 (2.5)		<.001	
	Moderate PA (hours/week)	0.5 (1.3)		0.7 (2.3)		0.2 (2.6)		.47		0.5 (1.1)			0.4 (0.8)		–0.1 (1.4)		.27	
	Walking (hours/week)	0.6 (1.1)		1.0 (2.0)		–0.4 (2.2)		.05		0.5 (0.6)			0.7 (1.4)		–0.2 (1.5)		.10	
	Sedentary behavior (hours/week)	10.7 (7.8)		8.3 (4.9)		–2.4 (7.5)		<.001		11.8 (8.3)			9.7 (6.9)		–2.1 (5.7)		<.001	
	Total PA (minutes/week)	123.4 (209.0)		250.5 (535.3)		127.1 (574.6)		.02		117.2 (123.9)			161.7 (204.3)		44.4 (224.8)		.04	
	Steps (n/day)	6224.2 (2048.5)		9214.3 (3205.5)		2990.1 (2892.5)		<.001		6272.1 (2024.5)			8945.5 (3843.3)		2673.4 (3124.6)		<.001	
**MetS^h^ factors**																		
	Number, mean (SD)	2.7 (1.0)		2.0 (1.1)		–0.7 (1.1)		<.001		3.0(1.0)			2.1(1.1)		–0.8 (1.1)		<.001	
	0, n (%)	0 (0.0)		12 (10.6)		N/A^i^		N/A		0(0.0)			8(7.5)		N/A		N/A	
	1, n (%)	16 (14.2)		25 (22.1)		N/A		N/A		10(9.2)			24(22.2)		N/A		N/A	
	2, n (%)	35 (31.0)		34 (30.1)		N/A		N/A		25(23.1)			36(33.3)		N/A		N/A	
	3, n (%)	32 (28.3)		33 (29.2)		N/A		N/A		33(30.6)			25(23.1)		N/A		N/A	
	4, n (%)	26 (23.0)		9 (8.0)		N/A		N/A		33(30.6)			14(13.0)		N/A		N/A	
	5, n (%)	4 (3.5)		0 (0.0)		N/A		N/A		7(6.5)			1(0.9)		N/A		N/A	

^a^SBP: systolic blood pressure.

^b^DBP: diastolic blood pressure.

^c^WC: waist circumference.

^d^HDL-C: high-density lipoprotein cholesterol.

^e^TG: triglyceride.

^f^HBA_1c_: glycated hemoglobin.

^g^PA: physical activity.

^h^MetS: metabolic syndrome.

^i^N/A: not applicable.

### Changes in the Number of Steps to Meet Guidelines 

Table 3 shows the changes in the number of steps before and after the intervention. For the prevention of chronic diseases, according to the guidelines for PA in Korea and the United States, taking at least 10,000 steps per day is recommended [28]. In the enhanced intervention group, there was a significant increase in the number of participants that met the recommended step count after the intervention. Although this number also increased in the standard intervention group, the change was not statistically significant. Table 3 shows the monthly average number of steps from baseline, demonstrating no significant differences in the initial step counts between the two intervention groups, with the counts increasing in both groups until the fourth month. In the enhanced intervention group, the number of steps continued to increase from the beginning to the end of the trial, whereas in the standard intervention group, the number of steps gradually decreased from the fifth month until the last month.

**Table 3:** Proportion of participants meeting the step guidelines before and after the intervention.

Steps per day			Baseline		Follow-up	*P* value	
**Enhanced intervention group (n=113), n (%)**							.002
	<10,000	104 (92.0)		87 (76.9)			
	≥10,000	9 (8.0)		26 (23.1)			
**Standard intervention group (n=108), n (%)**							.72
	<10,000	100 (92.6)		92 (85.2)			
	≥10,000	8 (7.4)		16 (14.8)			

## Discussion

### Principal Findings and Comparison With Prior Studies

The results of this study showed that the app of the wearable device itself was effective in reducing blood pressure, waist circumference, and HbA_1c_. The addition of a mobile app along with the wearable device to the intervention also led to reduced weight and BMI. This result is consistent with a previous study that used a mobile app and a wearable device in an overweight cohort, and found that increasing PA reduced the risk of type 2 diabetes and lowered blood pressure through clinically significant weight loss [29]. In addition, the results of a study measuring the number of steps and activity levels in the elderly were similar to those obtained in the enhanced intervention group of this study regarding significant reductions in their weight and BMI [30]. Rowley et al [31] also found that an intervention in which participants were provided feedback via websites, along with education and self-management had more activity than that of the group that received only an activity meter. These results are attributed to the additional education on MetS disease, normal reference values, correct eating habits, and the importance of increased activity. However, this result is in contrast to the findings of another study showing that monitoring and smartphone apps did not significantly improve PA in patients with chronic obstructive pulmonary disease [32].

Increased PA has been shown to prevent or delay the onset of diabetes, heart disease, and chronic diseases [33]; thus, increasing the PA among groups with at least one major risk factor should be considered a priority for disease prevention.

In this study, the IPAQ was used to evaluate the aspects of PA that cannot be measured using wearable devices alone, while a wearable device was used to quantify movement data to assess PA. Accordingly, the number of steps was measured using wearable devices, and the amount of personal PA, including vigorous exercise and sitting time, was investigated by the IPAQ. Text messages and mobile apps are currently being used as interventions to increase PA owing to their affordability and convenience, and have been established as important strategies that can change the lifestyle of the wearer. However, few studies have investigated how wearable devices and apps can be used to prevent MetS through changes in PA in the middle-aged population living in rural areas of Korea. Wang et al [34] reported that using a wearable device and providing a text message for 6 weeks resulted in participants with obesity significantly increasing their activity by 1266 steps in 1 week; however, this was reduced to a mere increase of 24 steps in the consecutive weeks. Additionally, the total PA time decreased by 15 minutes. In this study, the number of steps increased by 2990 by the end of the clinical trial, and the total PA time increased by 127 minutes. In previous studies, a minimum of 150 minutes of moderate to vigorous physical activity (MVPA) per week was recommended to prevent cardiovascular disease and chronic diseases, which is also the recommended PA level in the Korean and US guidelines [7,35,36]. In this study, only the enhanced intervention group reached the recommended MVPA (188 minutes per week vs 118 minutes per week in the standard intervention group). Owolabi et al [37] reported that adherence to the recommended PA was low even after a text message intervention; however, in this study, intervention through the app and text messages had an effect on participants reaching the recommended goal. In addition, the enhanced intervention group showed higher persistence, whereas in the standard intervention group, the number of steps initially increased and then decreased from the middle to late part of the study period.

### Recommendations and Limitations

To our knowledge, this is one of the few studies that analyzed the impact of wearable devices on the promotion of PA in middle-aged people living in rural areas of Korea. Recently, the accumulation of clinical data through connected devices with mobile apps has soared. However, evaluation of the clinical measures obtained by using the intervention service has not yet reached the pace of development. In both the enhanced intervention group using wearable devices and mobile apps and the standard intervention group using only wearable devices, the PA of the participants improved. This result suggests that the improvement of PA in the standard intervention group may have been a psychological effect of participating in the study. However, the knowledge and information provided in text messages and the app served as a major factor in preventing MetS. The distribution of participants without any MetS factors was confirmed to increase by 12 (10.6%) among the 113 participants in the enhanced intervention group and by only 8 (7.5%) among the 108 participants in the standard intervention group. This finding is in contrast to the study by Jakicic et al [38], who found that the protocol for monitoring PA and providing feedback did not have significant effects. According to Patel et al [39], wearable devices can promote health behavior change, but their successful use and potential health benefits depend more on the design of engagement strategies than on the characteristics of the technology. This highlights the need for personal encouragement, competitive spirit and collaboration, and effective feedback that are linked to human behavior.

A limitation of this study is that the participants were only recruited from rural areas in Korea; hence, the cohort was fairly homogeneous with a similar lifestyle and infrastructure. In the future, it will be necessary to provide information on cultural factors and rural geographical characteristics suitable for Koreans, and that proper diet and lifestyle modifications are needed to prevent chronic diseases. In addition, comparative studies between urban and rural residents in Korea are needed, along with examining the trends of lifestyle and clinical indicators, including various diseases and patient groups, through various wearable devices and advanced mobile apps.

### Conclusions

PA monitoring and intervention using a wearable device for 6 months effectively prevented MetS in rural participants in Korea. Moreover, blood pressure, waist circumference, and HbA_1c_ levels improved in both intervention groups, which were effective in reducing MetS factors. However, there was a difference in the persistence of PA between the two groups. The enhanced intervention group continuously increased the amount of PA above the recommended level to prevent MetS, and as a result, body weight and BMI were further improved. Since the clinical values that can confirm the improvement of MetS may not improve in a short time, a longer-term study is needed.

## Appendix

Multimedia Appendix 1 CONSORT-eHEALTH checklist (V 1.6.1).

## Abbreviations

HbA_1c_: glycated hemoglobin
IPAQ: International Physical Activity Questionnaire
MET: metabolic equivalents of task
MetS: metabolic syndrome
mHealth: mobile health
MVPA: moderate to vigorous physical activity
PA: physical activity
